# Neuroprotective properties of erythropoietin (Epo) and its receptor (EpoR) in open spinal dysraphism (OSD): an investigation of EpoR expression in a rat OSD model, along with *in vitro* studies on the neuroprotective effects of Epo on rat spinal cord-derived neural progenitor cells

**DOI:** 10.1007/s00381-025-07032-8

**Published:** 2025-11-17

**Authors:** Friederike Knerlich-Lukoschus, Jacqueline Clüver, Inez Manczuk, Frieda Bayler, Dana Hellmold, Michael Synowitz, Janka Held-Feindt

**Affiliations:** 1 Division of Pediatric Neurosurgery, Department of Neurosurgery, University Medical Center Göttingen, Robert-Koch-Str. 40, 37075 Göttingen, Germany; 2 Department of Neurosurgery, University Hospital of Schleswig-Holstein Campus Kiel, Arnold-Heller-Str. 3, House D, 24105 Kiel, Germany

**Keywords:** Myelomeningocele, Neuroprotection, Animal model, Amniotic fluid, Neural progenitor cell culture

## Abstract

**Purpose:**

Research into treatment options that complement surgery for open spinal dysraphism (OSD) is necessary to further improve outcomes following prenatal or postnatal surgery. We investigated erythropoietin (Epo) and its receptor (EpoR) as a potential neuroprotective agent and target in OSD.

**Methods:**

Epo- and EpoR-expression was examined on mRNA and immunohistochemical level in neuroplacodes obtained from a rat retinoic acid-induced OSD model at E16, E18, E22. Neural progenitor cells (NPCs) derived from spinal cords of adult Long Evans rats were exposed to varying concentrations of human artificial amniotic fluid (aAF). Cytotoxicity assays were conducted to assess the impact of aAF exposure, with and without Epo. The influence of Epo on NPC’s cellular marker expression was analyzed using qRT-PCR.

**Results:**

EpoR mRNA expression was elevated significantly on E18 and E22 in OSD- compared to control tissue. EpoR immunoreactivity exhibited consistent expression in ventral and dorsal horns, central canal, and ganglia. It co-stained with Hif-2α, β-III-tubulin, NeuN, and Musashi1. Exposure of NPCs to aAF reduced proliferation and increased death rates dose-dependently. Adding Epo significantly counteracted antiproliferative and cytotoxic aAF effects on NPCs, resulting in similar or lower death rates than those observed in untreated controls. Exposure of NPCs to aAF reduced their expression of neuronal markers. Adding Epo restored their differentiation capacities.

**Conclusion:**

Induction of EpoR in neuroplacodes, its co-staining with neuronal and NPC markers, and the neuroprotective effects of Epo on aAF-treated NPCs in vitro establish Epo as a promising candidate for neuroprotective therapies that can supplement surgical measures for OSD.

**Supplementary Information:**

The online version contains supplementary material available at 10.1007/s00381-025-07032-8.

## Purpose

Open spinal dysraphism (OSD) is one of the leading causes of disability and mortality in children worldwide [1]. Significant advances have been made in the surgical and medical care of OSD patients, including developing fetal surgical approaches that preserve motor function [2]. However, long-term problems remain [3]. As surgical improvement cannot solve all OSD-associated problems [4], further treatment options must be explored to improve outcomes of pre- or postnatal surgeries.

According to the “two-hit hypothesis” [1, 2], toxic substances in the amniotic fluid and mechanical impacts activate similar signaling pathways in [4, 5] neuroprotective strategies may be suitable for OSD. Recombinant human erythropoietin (Epo) protects against neurodegenerative processes in neurological disorders [6]. Its “robust neuroprotective properties” [7], its clinical applicability in neonates [8–11], and the fact that its receptor (EpoR) is induced after SC injury in anatomical regions associated with neurorestorative processes [12], make Epo interesting for OSD. Since the neuroprotective potential of Epo in OSD has not yet been investigated, these studies focus on this molecule.

We investigated EpoR induction and expression patterns in neuroplacodes obtained from our retinoic acid (RA)-induced OSD rat model at different fetal times, along with EpoR-regulating molecules, hypoxia-inducible factors (Hifs). After identifying EpoR as a potential neuroprotective target in OSD, we investigated the neuroprotective effects of its ligand on rat SC-derived neural progenitor cells (NPCs) in vitro: NPCs exposed to artificial amniotic fluid (aAF) with or without Epo were analyzed for proliferation, cell death rates, and gene expression profiles (see Supplementary Fig. 1 for Graphical Abstract).

## Materials and methods

### RA-OSD rat model

The RA-OSD rat model, introduced by Danzer et al. [13], was used. 15 pregnant rats received RA, and 5 received olive oil only. Littermates were randomized across experimental groups. For further details on the rat paradigm, we refer to [14].

For mRNA isolation, dysraphic OSD defects were separated from intact SC sections, snap-frozen in liquid nitrogen, and stored at −80 °C. Corresponding SC segments of control animals were handled in the same way. For histological studies, fetuses were fixed in 4% paraformaldehyde/phosphate-buffered saline (pH 7.4) for ten days, embedded in paraffin wax, and sectioned (0.5 µm thickness).

Protocols were approved by the Institutional Animal Care and Use Committee of the University of Schleswig–Holstein, Campus Kiel, Germany (reference number: V242-22,800/2016 (39–3/16). Animal experiments were carried out in compliance with the European Union directive on animal protection used for scientific purposes.

### Immunohistochemistry

Details on immunohistochemical procedures have been published previously [15, 16]. Cellular profiles of EpoR immunoreactivity (IR) were investigated by double-labeling with standard antibodies against neuronal and glial cells. Details on antibodies are summarized in Table 1. Controls included: Omitting the primary antibody and staining with monoclonal rabbit IgG (R&D Systems, Wiesbaden-Nordenstadt, Germany, #AB-105-C). Secondary antibodies were labeled with Alexa Fluor 488 (1:1,000) or Alexa Fluor 555 (1:1,000). Nuclei were stained with 4',6-diamidino-2-phenylindole (DAPI) (Molecular Probes/Invitrogen, Life Technologies, Karlsruhe, Germany; 1:30,000). Fluorescence was visualized using a Zeiss microscope Observer.Z1 + ApoTome.2, with an AxioCam MRm camera (Software: ZEN Pro [2012], Carl Zeiss GmbH, Jena, Germany). Qualitative Immunohistochemical analyses were conducted double blinded.

**Table 1:** **A:** Identifiers of TaqMan assays with gene-specific primers and probes, **B:** Applied antibodies in immunohistochemical procedures, **C:** Applied antibodies in Western Blot analysis

(A) TaqMan primer probes used for real-time RT-PCR (Applied Biosystems, Foster City, CA)				
*Gene*	*Specificity*		*Sequence*	
GAPDH	Glycerinaldehyd-3-phosphat-Dehydrogenase (housekeeping gene)		Rn99999916_s1	
Epo	Erythropoietin		Rn01481376_m1	
EpoR	Erythropoietin receptor		Rn00566533_m1	
HIF1a	Hypoxia inducible factor 1		Rn01472831_m1	
HIF2a	Hypoxia inducible factor 2		Rn00576515_m1	
beta-III-tubulin	Marker for early neuronal differentiation		Rn01431594_m1	
MAP	Microtubule-associated protein 2		Rn00565046_m1	
Musashi1	Neural RNA-binding protein, strongly expressed in fetal and adult neural stem cells		Rn00596059_m1	
Ki67	Proliferation marker		Rn01451446_m1	
(B) Antibodies used for Immunohistochemistry				
*Marker*	*Specificity*	*Dilution*	*Source*	*RRID*
Epo	Erythropoietin (intracellular, cell surface, extracellular localization)	Polyclonal rabbit IgG, 1:250	Abcam Cambridge, UK; #ab226956	n.a
EpoR	Erythropoietin Receptor (intracellular, cell surface localization)	Polyclonal rabbit IgG polyclonal, 1:50,	Abcam Cambridge, UK; #ab244202	n.a
HIF1a	Hypoxia-inducible factor 1 (cytoplasm, cell nuclei)	Polyclonal rabbit IgG, 1:100	Novus Biologicals Abingdon, UK; #nb100-134	AB_350071
HIF2a	Hypoxia-inducible factor 1 (cytoplasm, cell nuclei)	Polyclonal rabbit IgG, 1:100	Novus Biologicals Abingdon, UK; #nb100-122	AB_10002593
b-III-tubulin	Marker for early neuronal differentiation (cytoplasm)	Polyclonal rabbit IgG, 1:800	Sigma-Aldrich, Schnelldorf, Germany #T2200	AB_262133
NeuN	Neuron specific nuclear protein (neuronal nuclei)	Monoclonal mouse IgG, 1:500	Milipore, Schwalbach, Germany/#MAB377	AB_2298772
Musashi1	Marker for fetal and adult neural stem cells (cytoplasm, nuclei)	Monoclonal mouse IgG, 1:1000	R&D Systems, Wiesbaden-Nordenstadt, Germany #MAB2628	AB_2235632
(C) Antibodies used for Western Blot				
*Marker*	*Dilution*	*Source*		*RRID*
PCNA (Proliferating-Cell-Nuclear-Antigen)	Monoclonal mouse IgG, 1:1000	Cell Signaling, Massachusetts, USA #2586		AB_2160343
pSTAT5 Tyr694 (Signal transducer and activator of transcription 5)	Polyclonal rabbit IgG, 1:250	Cell Signaling, Massachusetts, USA #9351		AB_2315225
p-Akt Thr308 (Protein kinase B)	Polyclonal rabbit IgG, 1:200	Cell Signaling, Massachusetts, USA #9275		AB_329828
Hsp90 α/β (Heat shock protein 90)	Polyclonal rabbit IgG, 1:1000	Santa Cruz, Dallas, Texas, USA, #sc-7947		AB_2121235
Donkey anti mouse (HRP-coupled Secondary antibody)	1:10.000	Invitrogen ThermoFisher, Massachusetts, USA A16011		AB_2534685
Donkey anti rabbit (HRP-coupled Secondary antibody)	1:12.500	Invitrogen ThermoFisher, Massachusetts, USA A16035		AB_2534709

### Cultivation of NPCs

NPCs were generated from adult male Long Evans rats’ SC (details have been published previously [16]). All protocols and procedures were approved by the University of Schleswig–Holstein's Institutional Animal Care and Use Committee (Ministerium für Landwirtschaft, Umwelt und ländliche Räume des Landes Schleswig–Holstein; 2005, 2007, 2008 [V 312–72,241.121–35(34–4/05)], and 2009 [V 312–72,241.121–35(77–6/09)]). The obtained neurosphere-like cells were cultured as described before [17]. NPCs were identified by their ability to form neurospheres, survive and proliferate under stem cell conditions, and differentiate into mature cell types [17].

### Preparation of artificial in vivo-adapted amniotic fluid (aAF)

aAF was prepared based on the mean concentrations reported in the literature to closely replicate the physiological composition of human AF at 22 weeks of gestation [18–22]. EMEM (#M0325, ThermoFisher Scientific) and F12 (#31,765, ThermoFisher Scientific) were selected as base media in equal proportions (1:1). The concentrations of added components were adjusted to align with the reported values to ensure physiological relevance. The final concentration, details of the supplemented components, pH, and osmolarity are listed in Table 2.

**Table 2:** Supplementation of salts and amino acids to create artificial Amniotic fluid

Component	F12/EMEM (1:1) [mM]	Literature Mean ± SD [mM]	Supplementation [mM]
Sodium	52,360	135,4 ± 2,19	44,53
Potassium	2,18	3,8 ± 0,18	1,62
Chloride	78,18	106,9 ± 4,90	27,75
Calcium	0,38	1,5 ± 0,22	0,43
Magnesium			0,187
Glucose	7,78	44,1 ± 5,51	33,6
Urea		23,5 ± 0,49	23,5
Glycine	0,05	0,19 ± 0,001	0,13
L-Alanine	0,05	0,31 ± 0,014	0,25
Glutamine	0,19	0,22 ± 0,001	0,03
Arginine	0,56	0,03 ± 0,007	
Asparagine	0,51	0,00	
Aspartic acid	0,057	0,02	
Cysteine	0,066	0,06 ± 0,013	
L-Glutamic Acid	0,11	0,07 ± 0,033	
Glutamine	0,20	0,22 ± 0,001	
Histidine	0,07	0,05 ± 0,001	
Isoleucine	0,04	0,03 ± 0,011	
Leucine	0,08	0,07 ± 0,027	
Lysine	0,13	0,13 ± 0,021	
Methionine	0,02	0,01 ± 0,003	
Phenylalanine	0,03	0,04 ± 0,011	
Proline	0,15	0,12 ± 0,012	
Serine	0,05	0,08 ± 0,035	
Threonine	0,07	0,13 ± 0,011	
Trypthophan	0,01	0,00	
Tyrosine	0,04	0,04 ± 0,011	
Valine	0,07	0,09 ± 0,031	
Ornithine	0,00	0,05 ± 0,009	
Taurine	0,000	0,12 ± 0,009	
pH	7.5–7.8		
Osmolarity	300.66 mOsm/kg		

*SD* Standard Deviation, *mM* millimole. Distributor information: Sodium bicarbonate (Sigma Aldrich, Germany; S5761-500 g), sodium citrate (Carl Roth GmbH & Co. Karlsruhe, Germany; ≥ 99% p.a. ACS #3580.1), calcium chloride (Carl Roth; ≥ 95% water-free; #A119.1), magnesium chloride (Carl Roth; ≥ 99% water-free; #KK36.1), potassium chloride (Merck KGaA, Darmstadt, Germany; EMSURE® for analysis; #1.04936.0500), sodium chloride (Carl Roth; ≥ 99,5% p.a. ACS, ISO; #3957.1), Urea (Carl Roth; ≥ 99,5% Ph. Eur.Crist.; #X999.2), D-(+)-Glucose (Carl Roth; p.a. ACS; #X997.2), Glycine (Carl Roth; Pufferan® ≥ 99% p.a.; #3908.2), L-Alanine (Carl Roth; ≥ 98,5% for Biochemistry; #3076.1), and Glutamine (Carl Roth; Cellpure® ≥ 99%; HN08.1)

The solution's pH was adjusted to 7.8–7.5 using a maximum of 6 mL/1L of 1 N HCl to avoid excessive chloride ion concentrations. After ensuring the solution was free of precipitates and pH-stable, it was sterilized through 0.2 µm filters and aliquoted into single-use tubes for storage at −30°C for up to 1 month, ensuring aAF stability and preventing the need for freeze–thaw events. The pH was revalidated before use.

### Stimulation of NPCs

NPCs were seeded in 6 well-plates with 50,000 cells/well in stem cell media supplemented with different ratios of aAF (e.g., 30% artificial amniotic fluid and 70% cell media) and cultivated at 37 °C and 5% CO_2_ for up to 8 days. In a first step, rat NPCs were stimulated with aAF at concentrations of 0%, 30%, 55%, 80% and 100% (diluted with the remaining portion of cell culture medium) to assess the influence on proliferation by determining cell numbers after 4 and 7 days of stimulation.

To evaluate the neuroprotective properties of Epo, 20 ng/ml Epo (rat; Sigma-Aldrich; #E8905; concentration within the established effective range for neural precursors [23–26] was added in the presence or absence of aAF in additional samples for up to 8 days. Cytotoxicity assays, Western Blots, and analysis of gene regulation (see below) were performed at different time points throughout the stimulation period. Based on our preliminary tests with aAF treatment of NPCs, aAF was added at concentrations of 55% to ensure sufficient cells remained for longitudinal analysis (too few cells remained under 100% stimulation).

Basal EpoR expression was verified by qRT-PCR in rat NPCs (ΔCT = 18.05, n = 4). We measured proliferation by determining cell numbers after 2, 4, and 7 days of stimulation to investigate whether Epo exerts a protective effect.

To evaluate the involved signaling pathways affected by Epo, cells were stimulated with a JAK2/STAT5/Akt signaling pathway inhibitor (AG490, 10 µM; InSolutionTM, Calbiochem #658,411) for 30 min prior stimulation with Epo. Epo-stimulation was performed for a further 15 min under inhibitor influence, in the presence or absence of aAF.

### Cytotoxicity assay

The cytotoxic effects were evaluated using the CytoTox-Fluor™ Cytotoxicity Assay (Promega, Walldorf, Germany; #G9260) following the manufacturer’s instructions as described before [27]. Cell viability and proliferation were assessed by counting viable cells at the designated time points. The percentage of dead cells was calculated as described before [27]. Growth rates were determined as the fold increase in viable cells relative to day zero of the treatment.

aAF cytotoxicity was assessed over 8 days, with measurements taken on days 1, 2, 3, 4, and 8. Cells were treated daily with 0% (vehicle control; cell media, referred to as “control”), 30%, 55%, or 80% aAF 24 h before performing cytotoxicity, and the results were expressed as the x-fold increase in dead cells relative to the unstimulated control.

### qRT-PCR

RNA was extracted from cells and tissues using either TRIzol® reagent (#15,596,026; Invitrogen, Carlsbad, CA, USA) or the ARCTURUS® PicoPure® RNA Isolation Kit (#15,295,033; Applied Biosystems, Foster City, CA, USA), following the manufacturers' protocols. DNase digestion, cDNA synthesis, and qRT-PCR were performed as described previously [26], using TaqMan primer probes (Applied Biosystems). Details of the gene-specific primers and probes are provided in Table 1. Threshold cycle (C_T_) values were determined, and ∆C_T_ values were calculated for each sample using the formula ∆C_T_ = C_T_ (gene of interest) − C_T_ (GAPDH). Samples with undetectable expression levels were excluded from mean expression calculations. Data are presented as either ∆C_T_ values or linearized ∆C_T_ values (2-C_T_). Gene expression regulation following stimulation (with aAF +/Epo) is shown as relative gene expression, which is calculated as a fold change in expression using the formula: n-fold expression = 2∆C_T_ (control) − ∆C_T_ (stimulus).

### Western blot

Western Blot analysis was performed as described before [28]. Briefly, treated cells were harvested in lysis buffer [28], and 15–20 µg protein per sample was used for Western blotting experiments [28]. Details on primary and secondary antibodies are summarized in Table 1. Equal protein loading was confirmed using Hsp90, as described previously [28].

### Statistical analysis

Statistical analyses were conducted using GraphPad Prism 8.4® software (GraphPad Software, San Diego, California, USA). Depending on the experimental design, a two-tailed Student's t-test or a two-way ANOVA was performed. Statistical significance is indicated with asterisks based on p-values: * *p* < 0.05, ** *p* < 0.01, and *** *p* < 0.001.

## Results

### EpoR expression and anatomical localization in OSD rat model-derived neuroplacodes

**Fig. 1:**
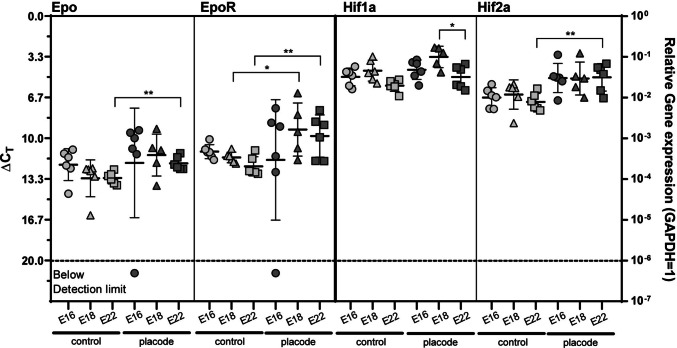
mRNA expression of Epo and EpoR and their regulators Hif-1α and Hif-2α in neuroplacodes obtained from the rat RA-OCD Model. (**A**) Epo, EpoR mRNA expression was detected at significantly elevated levels in the neuroplacodes at E18 (EpoR; * *p* < 0.05) and E22 (Epo/EpoR; ** *p* < 0.01) compared to the respective controls. (**B**) Unlike Hif-1α, Hif-2α mRNA was detectable at increased levels at E16, E18, and E22 compared to the control samples. At E22, these differences were statistically significant (** *p* < 0.01); n = 4–5 biological replicates; the significances were determined using a non-paired two-tailed *t*-test (* *p* < 0.05; ** *p* < 0.01; Epo Ctrl E22 vs. P E22 mean difference: −1.193, 95% CI: −1.847 – −0.539; EpoR Ctrl E18 vs. P E18 mean difference: −2.287, 95% CI: −4.319 – −0.257; EpoR Ctrl E22 vs. P E22 mean difference: −2.480, 95% CI: −4.206 – −0.753; Hif-1α P E18 vs. P E22 mean difference: 1.647, 95% CI: 0.409–2.884; Hif-2α Ctrl E22 vs. P E22 mean difference: −2.007, 95% CI: −3.209 – −0.805). Error bars correspond to the standard deviations. Abbreviations: RA-OCD = retinoic acid-induced open spinal dysraphism rat model; Epo = erythropoietin, EpoR = erythropoietin receptor, Hif = hypoxia inducible factor

Epo and EpoR mRNA were detectable in control and OSD placode tissue at E16, E18, and E22. EpoR mRNA was significantly higher in neuroplacodes at E18 and E22 compared to controls (Fig. 1). No differences were found in Epo mRNA detection between OSD and control groups. Since hypoxia-induced factors regulate Epo and EpoR expression [29, 30], we investigated whether similar EpoR induction factors might play a role in OSD by examining hypoxia-inducible factors Hif-1α and Hif-2α as indirect indicators of hypoxia-related mechanisms. Unlike Hif-1α, Hif-2α mRNA was detectable at increased levels at E16, E18, and E22 compared to controls. At E22, these differences were statistically significant (*p* < 0.01) (Fig. 1).

**Fig. 2:**
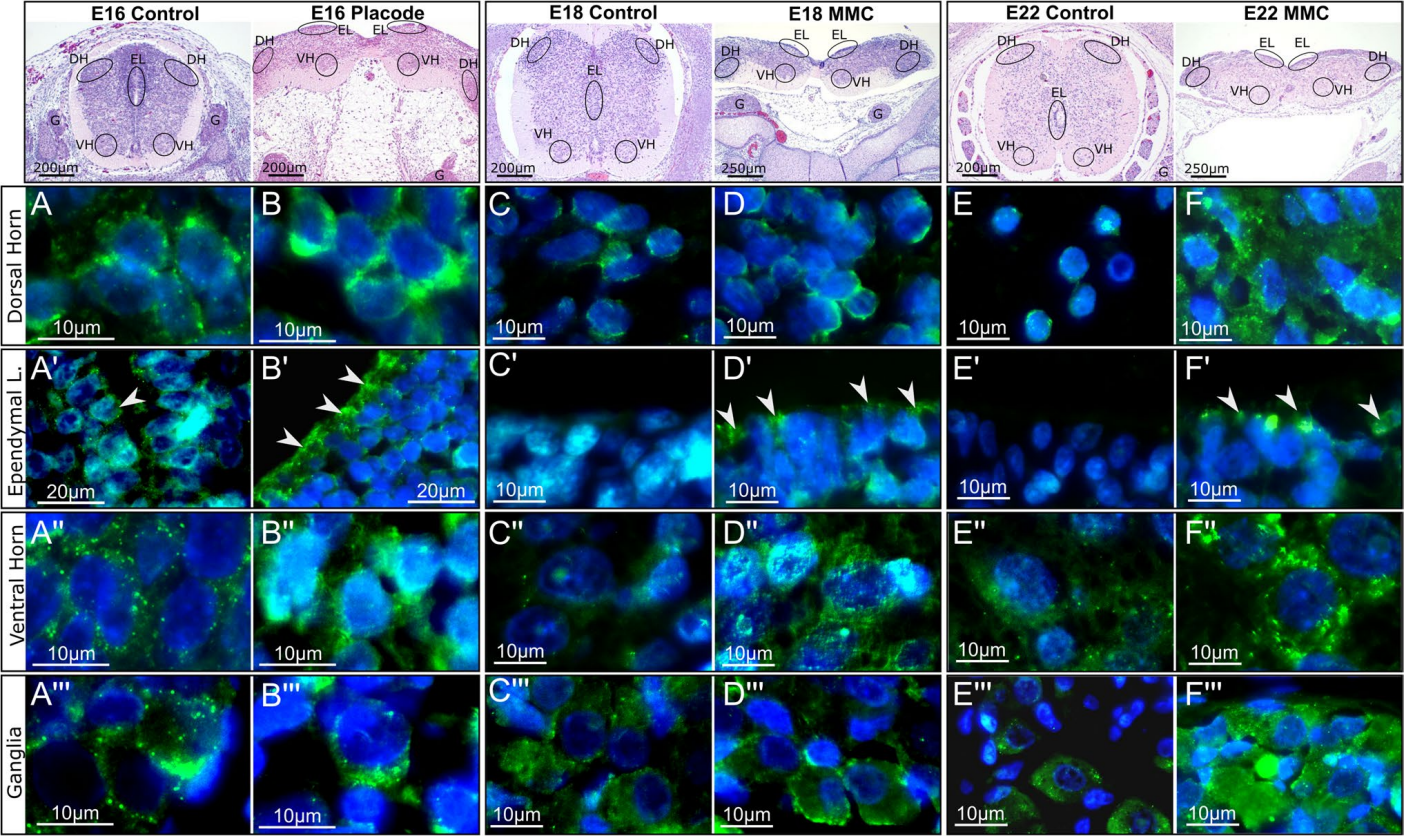
Anatomical expression patterns of EpoR immunoreactivity in spinal cord segments of control animals and neuroplacodes obtained from the OSD model. In the first row, the anatomical localizations in the respective spinal cord or neuroplacode hematoxylin/eosin-stained tissue sections are marked (DH = dorsal horn; EL = ependymal layer—central canal in control animals; matrix zone in neuroplacodes; VH = ventral horn, G = ganglia). The columns below each overview section show the EpoR immunoreactivity (green) in the respective anatomical region of control and neuroplacode tissues at E16 (A to A’’’ = control and B to B’’’ = neuroplacode), E18 (C to C’’’ = control and D to D’’’ = neuroplacode), E22 (E to E’’’ = control and F to F’’’ = neuroplacode). Arrowheads depict EpoR staining in the ependymal layer. Scale bars are provided in each figure. n = 3 biological replicates (animals) with n = 6 technical replicates (serial sections)

In control tissues, EpoR-immunoreactive cells were consistently detected in the dorsal and ventral horns and ganglia at all examined time points, and in the central canal ependymal layer on E16 (Fig. 2). In OSD placodes, EpoR was also detected in these regions. Additional EpoR expression was observed on E18 and E22 in the outer layer of the dysraphic placode (matrix zone), which corresponds to the central canal ependymal layer (Fig. 2B’, D', F’).

**Fig. 3:**
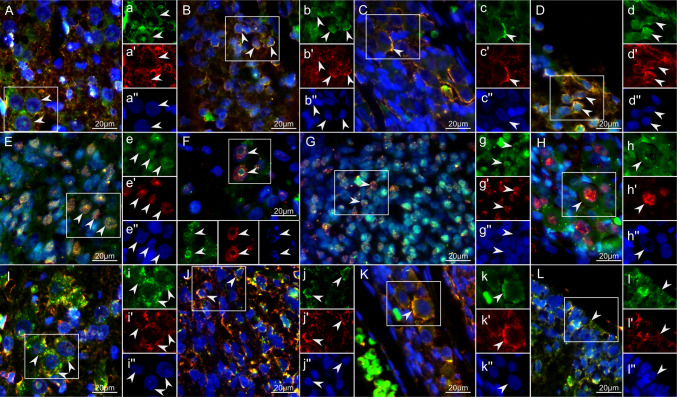
EpoR co-staining with neuronal and progenitor markers in neuroplacode tissue obtained from the rat RA-OSD model. In all figures, EpoR is shown in green, the cellular markers in red, and DAPI for labeling the cellular nuclei in the blue channel. White rectangles in the merged images depict the areas for which separated channels are provided (labeled with lowercase letters). A – D: EpoR co-staining with β-III-tubulin in the placode at E22 in the ventral Horn (**A**), dorsal horn (**B**), spinal cord ganglia (**C**), and ependymal layer (**D**). E–H: EpoR co-staining with NeuN in the ventral horn at E18 (**E**), in the ventral horn at E22 (**F**), dorsal horn (**G**), and dorsal ganglia (**H**). I – L: EpoR co-staining with Musashi 1 at E22 in the ventral horn (**I**), dorsal horn (**J**), ganglia (**K**), and ependymal layer (**L**). Scale bar in all figures = 20 µm. n = 3 biological replicates (animals) with n = 6 technical replicates (serial sections)

Epo staining patterns were similar to those of its receptor (data not shown). In the anatomical regions described, EpoR-immunoreactive cells mainly exhibited morphological characteristics of neuronal cells. Double-labeling confirmed EpoR co-staining with markers for early (β-III-tubulin) (Fig. 3A to D) and mature neuronal cells (NeuN) (Fig. 3E to H). EpoR also co-stained with Musashi1, a marker for NPCs (Fig. 3I to L). Other cell markers, such as nestin, BLBP, GFAP, and NG2, were also detectable in the described anatomical regions. However, as these markers were not co-stained with EpoR-immunoreactivity, they were not pursued in the present study.

**Fig. 4:**
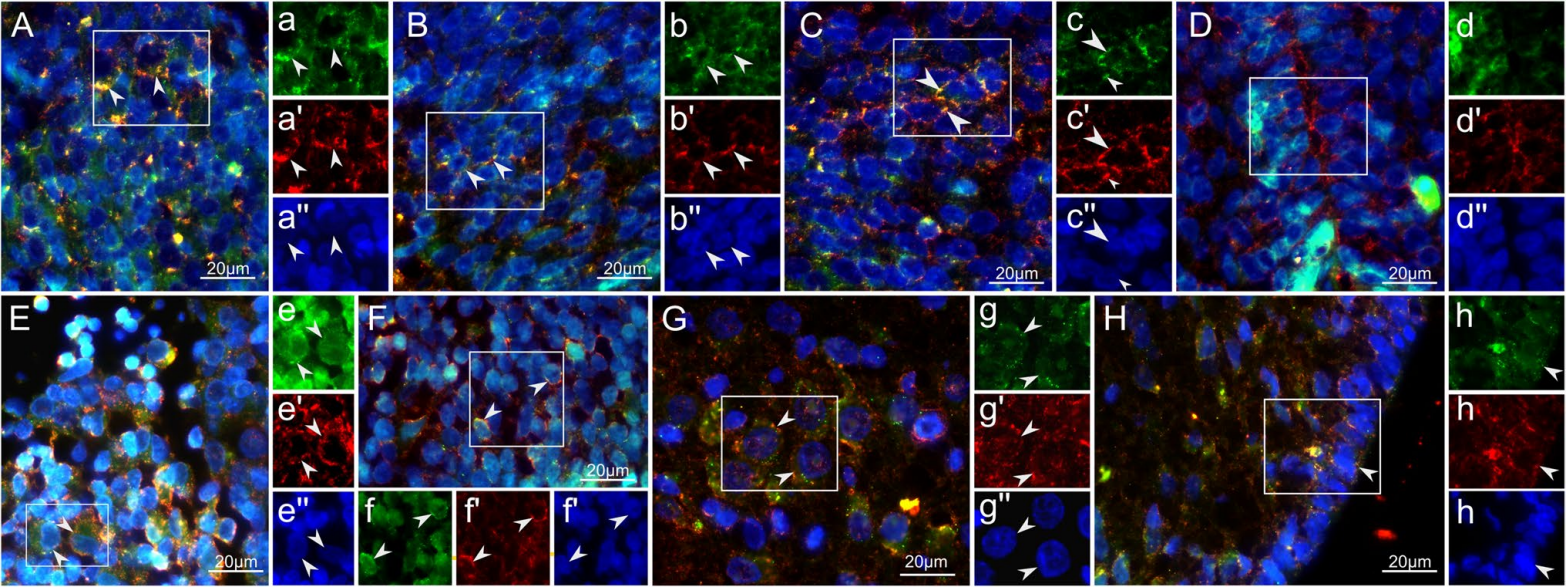
EpoR co-staining with HIF-2α in neuroplacode tissue obtained from the rat RA-OSD model. In all figures, the following color channels were applied: EpoR green, Hif-2α red, and DAPI (labeling the cellular nuclei) blue. White rectangles in the merged images depict the areas for which separate channels are provided (labeled with lower case letters); Yellow/orange indicates co-staining of EpoR- and Hif-2α-immunoreactivity. (**A**) EpoR co-staining with Hif-2α in dorsal ganglia of control spinal cord (SC) specimen at E16. (**B**) EpoR co-staining with Hif-2α in the dorsal horn of the control SC specimen at E16. (**C**) EpoR co-staining with Hif-2α in the ventral horn of a control SC specimen at E16. (**D**) EpoR co-staining with Hif-2α in the central canal of the control SC specimen at E16. (**E**) EpoR co-staining with Hif-2α in dorsal ganglia at the level of the neuroplacode of RA-OSD derived specimen at E16. (F) EpoR co-staining with Hif-2α in the dorsal horn of the neuroplacode of RA-OSD-derived specimens at E16. (**G**) EpoR co-staining with Hif-2α in the ventral horn of the neuroplacode of RA-OSD-derived specimens at E22. (**H**) EpoR co-staining with Hif-2α in the ependymal layer of the neuroplacode of RA-OSD-derived specimens at E22. Scale bar in all figures = 20 µm. *n* = 3 biological replicates (animals) with n = 6 technical replicates (serial sections)

Qualitative double staining of EpoR and Hif-2α confirmed co-staining of cells in the aforementioned anatomical areas (Fig. 4).

### Influence of aAF on NPCs’ proliferation and cell death rates

**Fig. 5:**
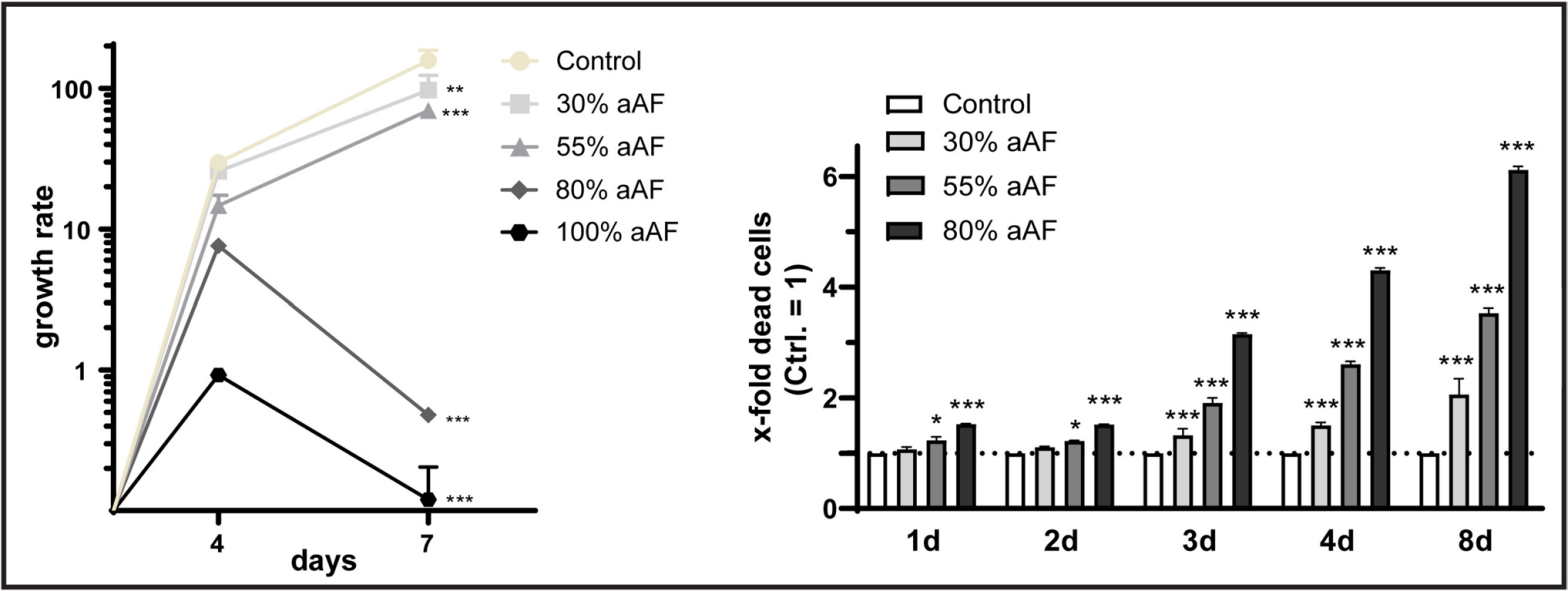
Effect of artificial amniotic fluid (aAF) on neural progenitor cells (NPCs). Left: Stimulation with aAF resulted in an anti-proliferative effect, which increased progressively with higher aAF concentrations; *n* = 4 biological replicates. Right: aAF resulted in higher numbers of dead cells in a concentration-dependent manner; *n* = 2 biological replicates with *n* = 2 technical replicates each; the significances were determined using an ordinary two-way ANOVA test followed by a Tukey’s multiple comparison test (* *p* < 0.05; *** *p* < 0.001; Left: Ctrl vs. 30% aAF mean difference: 60.800, 95% CI: 19.370–102.200; Ctrl vs. 55% aAF mean difference: 88.470, 95% CI: 47.040–129.900; Ctrl vs. 80% aAF mean difference: 157.9, 95% CI: 111.600–204.200; Right: 1 d Ctrl vs. 55% aAF mean difference: −0.235, 95% CI: −0.447 – −0.023; 1 d Ctrl vs. 80% aAF mean difference: −0.525, 95% CI: −0.737 – −0.3126; 2 d Ctrl vs. 55% aAF mean difference: −0.220, 95% CI: −0.432 – −0.008; 2 d Ctrl vs. 80% aAF mean difference: −0.518, 95% CI: −0.729 – −0.305; 3 d Ctrl vs. 30% aAF mean difference: −0.325, 95% CI: −0.537 – −0.113; 3 d Ctrl vs. 55% aAF mean difference: −0.910, 95% CI: −1.122 – −0.698; 3 d Ctrl vs. 80% aAF mean difference: −2.153, 95% CI: −2.365 – −1.940; 4 d Ctrl vs. 30% aAF mean difference: −0.503, 95% CI: −0.715 – −0.290; 4 d Ctrl vs. 55% aAF mean difference: −1.610, 95% CI: −1.822 – −1.398; 4 d Ctrl vs. 80% aAF mean difference: −3.305, 95% CI: −3.517 – −3.093; 8 d Ctrl vs. 30% aAF mean difference: −1.063, 95% CI: −1.275 – −0.850; 8 d Ctrl vs. 55% aAF mean difference: −2.533, 95% CI: −2.745 – −2.320; 8 d Ctrl vs. 80% aAF mean difference: −5.118, 95% CI: −5.330 – −4.905). Error bars correspond to the standard deviation

30% and 55% aAF inhibited NPCs proliferation, while 80% and 100% aAF significantly reduced it (Fig. 5, left panel). The apparent antiproliferative effect of aAF suggested simultaneous cell death. Subsequent cytotoxicity assays confirmed this. The following experiments were performed with 30%, 55% and 80% aAF to ensure enough cells for longitudinal analysis (with 100% aAF stimulation, too many cells died). Cytotoxicity increased significantly from day 1 at 55% and 80% aAF, becoming more pronounced over time. By day 8, 80% aAF caused around six times more dead cells than the control. 55% aAF caused three and a half times more. Cytotoxicity at 30% aAF began on day 3 and increased gradually (Fig. 5, right panel).

### Influence of Epo on aAF-induced effects on NPCs

**Fig. 6:**
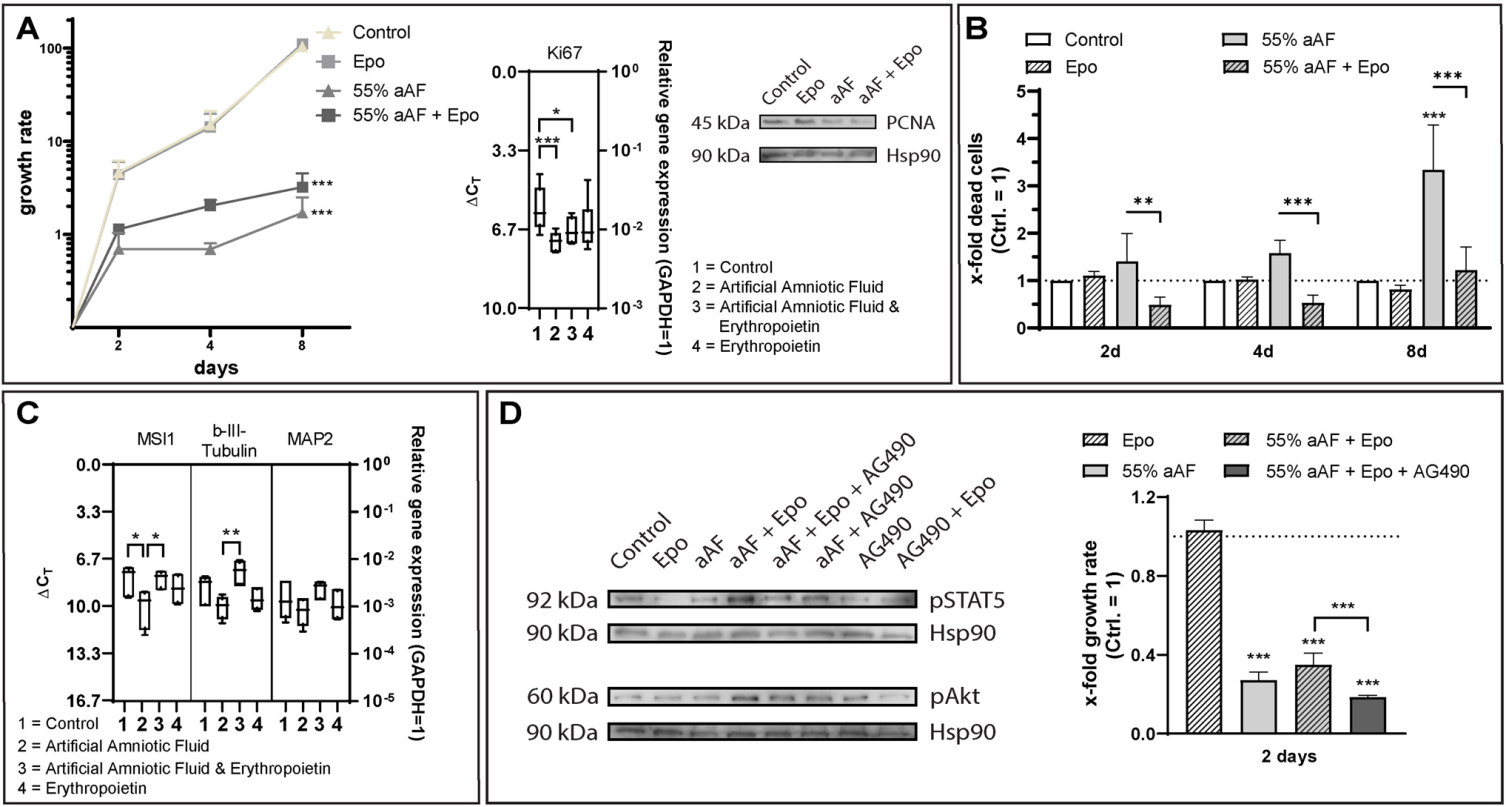
Effect of erythropoietin (Epo) on artificial amniotic fluid (aAF)-treated neural progenitor cells (NPCs). Results were expressed as the x-fold increase in dead cells relative to the unstimulated control. (**A**) Stimulation with aAF (55%) resulted in a pronounced antiproliferative effect compared to unstimulated control. Stimulation with aAF (55%) combined with Epo (20 ng/mL) resulted in less pronounced antiproliferative effects by aAF; growth rate: n = 6 biological replicates, Western Blot: n = 2 biological replicates, qPCR analysis: n = 3 biological replicates. (**B**) The cytotoxicity of aAF was counteracted by Epo stimulation; n = 6 biological replicates. (**C**) Rat progenitor cells stimulated with aAF showed a reduced expression of neuronal cell markers. Stimulation with aAF and Epo showed a tendency to restore gene expression to control levels; n = 3 biological replicates with n = 2 technical replicates each. (**D**) Western blotting confirmed robust phosphorylation of STAT5 and Akt mainly in response to Epo stimulation under aAF conditions, which was markedly attenuated with application of the inhibitor AG490 (10 µM); n = 2 biological replicates. The cell growth was lowest under aAF treatment, an effect that could be counteracted to a certain extent by Epo, which in turn was inhibited by the application of AG 490 (10 µM); n = 6 biological replicates. The significances (A + B + C) were determined using an ordinary two-way ANOVA test followed by a Tukey’s multiple comparison test (* *p* < 0.05; ** *p* < 0.01; *** *p* < 0.001; (**A**) Growth rate: Ctrl vs. 55% aAF mean difference: 102.9, 95% CI: 92.06–113.7; Ctrl. vs. 55% aAF + Epo mean difference: 101.4, 95% CI: 90.55–112.2 (**B**) 2 d 55% aAF vs 55% aAF + Epo mean difference: 0.9233, 95% CI: 0.204–1.643; 4 d 55% aAF vs 55% aAF + Epo mean difference: 1.043, 95% CI: 0.424–1.763; 8 d 55% aAF vs 55% aAF + Epo mean difference: 2.115, 95% CI: 1.396–2.834; 8 d Ctrl. vs 55% aAF mean difference: −2.340, 95% CI: −3.059 – −1.621 (C) MSI1 Ctrl vs. aAF mean difference: 2.042, 95%CI: 0.019–4.066; MSI1 aAF vs. aAF + Epo mean difference: −2.046, 95% CI: −4.070 – −0.023; b-III-Tubulin aAF vs. aAF + Epo mean difference: 2.490, 95% CI: 0.467–4.514). Furthermore, the significances (A + D) were determined using a non-paired two-tailed *t*-test (*** *p* < 0.001; (A) qPCR: Ki67 Ctrl. vs. 55% aAF mean difference: 1.425, 95% CI: 0.708–2.141; Ki67 Ctrl. vs. 55% aAF + Epo mean difference: 1.006, 95% CI: 0.250–1.761 (D) Ctrl. vs. 55% aAF mean difference: 0.728, 95% CI: 0.6913–0.7654; Ctrl. vs. 55% aAF + Epo mean difference: 0.650, 95% CI: 0.5963–0.704; Ctrl. vs. 55% aAF + Epo + AG 490 mean difference: 0.815, 95% CI: 0.806–0.825; 55% aAF + Epo vs. 55% aAF + Epo + AG490 mean difference: −0.160, 95% CI: −0.220—−0.111). Error bars correspond to the standard deviation

Stimulation with aAF (55%) resulted primarily in a pronounced antiproliferative effect compared to unstimulated control (Fig. 6A). The antiproliferative effects of 55% aAF stimulation were less pronounced in the presence of Epo (Fig.  6A, 55% aAF + Epo). Cytotoxicity Assay results showed that Epo application alone did not influence non-aAF-treated NPC cell death rates at any of the examined time points (Fig. 6B, Epo). The addition of Epo to 55% aAF maintained the levels of cell death below or comparable to those of the non-aAF-treated controls (Fig. 6B).

### Influence of Epo and aAF on NPC’s cell marker mRNA expression profiles

Figure 6C, panel 1, shows NPC’s mRNA expression level of Musashi1, b-III-tubulin, and MAP2 under basal conditions (1 = Ctrl = control). After two days of stimulation with 55% aAF, NPCs exhibited different expression patterns compared to untreated controls, with downregulation of precursor (e.g., Musashi1) and neuronal (e.g., β-III-tubulin, MAP2) markers (Fig. 6C, panels 2). Adding Epo to 55% aAF showed a tendency to restore the unstimulated control state (Fig. 6C, panel 3). Western blot analysis confirmed robust phosphorylation of STAT5, and Akt in response to Epo stimulation under aAF condition, which was markedly attenuated under JAK2/STAT5/Akt pathway inhibition via AG490 (10 µM). Furthermore, cell growth analysis revealed the lowest cell growth under aAF treatment, an effect that could be counteracted to a certain extent by Epo, which in turn was inhibited by the application of AG490 (Fig. 6D).

## Discussion

We investigated Epo as a potential candidate for neuroprotection in OSD. The prerequisite for this is given in principle with our detection of significantly increased EpoR mRNA expression in the neuroplacodes of our OSD model. This finding and the detectable EpoR immunoreactivity in ventral horns, which co-stained with neuronal and neural progenitor markers, encouraged us to investigate Epo’s effect on rat SC-derived NPCs, treated with aAF. This in vitro approach is based on the assumption that neurotoxic substances in the aAF, alongside mechanical effects, contribute to progressive damage to the exposed neuroplacode in OSD [1, 31]. It has been demonstrated that human AF exhibits cytotoxic effects on cells obtained from dissected rat SC [31, 32]. In surgically induced OSD, exchange of AF led to higher neural cell counts in MMC placodes obtained from chick embryos, compared to those in which the AF was not exchanged [33]. Our study confirmed similar, dose-dependent effects of aAF on rat SC NPCs, which exhibited significantly lower proliferation and higher cellular death rates when exposed to aAF. Our findings that adding Epo to aAF-treated NPC cultures significantly increased cell growth and decreased cell death rates via JAK2/STAT5/Akt-signaling suggest that Epo may play a protective role in mitigating the harmful effects of AF stimulation. This observation may be explained by Epo’s possible antiapoptotic effects, which have recently been demonstrated [34]. Given the increased apoptosis in OSD neuroplacodes, which contributes to the progressive loss of neural function in the late fetal period [35, 36], the antiapoptotic properties of EPO are interesting.

Our in vitro studies found that Epo showed the tendency to restore the expression pattern and, thus, the differentiation properties of NPCs: NPC cultures treated with aAF exhibited decreased mRNA expression levels of neuronal and neural progenitor cell markers. Adding Epo to these cultures tend to reverse this effect. Future studies will address the underlying molecular mechanisms of Epo's potential influence on NPC differentiation capacities.

Epo has been used in clinical trials to address various neuropathological conditions (see review [6]). In the pediatric clinical practice, Epo has been used in phase I studies of hypoxia-related neurological incidents in neonates [9–11]. Intravenously administering high doses of recombinant Epo achieved neuroprotective serum levels that were well tolerated by extremely low birth weight infants and did not cause excess morbidity or mortality [8]. These findings encourage the design of therapeutic trials using Epo as a neuroprotective molecule in other pathological conditions, such as OSD. Regarding the direct accessibility of the defect zone (i.e., exposed neuroepithelium) in OSD, one could consider applying Epo directly to the tissue during surgery. This could circumvent the problem of its short half-life and rapid clearance from the delivery site [37, 38]. Techniques for modifying tissue engineering scaffolds or hydrogels with Epo-loaded nanoparticles have been described in the literature [38–40]. When designing tissue-engineered scaffolds for OSD, it will be important to consider the interplay of different molecular mediators of the cascades induced by the second hit. Based on our previous findings regarding expression of proinflammatory cytokines in OSD neuroplacodes [14], tissue-engineered constructs that deliver a combination of immunomodulating and neuroprotective factors to the open dysraphic site could be a promising adjunct to existing fetal and early postnatal surgical techniques.

## Limitations

We used aAF with a concentration similar to 22 weeks of human gestation and neither considered the varying compositions of AF throughout the fetal period nor investigated the potential influence of individual AF substances on neural cells. Exposure to different concentrations of certain AF components, such as meconium, can have detrimental effects on the neuroepithelium in OSD [32]. Furthermore, we have prioritized maintaining the physiological Cl^−^ value over a pH adjustment to physiological ranges below pH 7.5, given the influence of Cl^−^ in nervous tissue and the critical effects of hyperchloremia. Therefore, the pH is slightly above physiological ranges. Another point is how EpoR expression is regulated in neuroplacodes, which remains unclear for now. The EpoR and Hif-2α mRNA expression levels alongside the co-staining of the molecules in neuroplacodes suggest that hypoxia-related mechanisms may also be involved in EpoR regulation in OSD, like in other conditions. Further comprehensive inhibition studies, including targeting downstream signaling cascades or the use of Epo analogues such as carbamylated Epo will be required to fully elucidate the pathways involved. The in vitro experiments were performed with adult rat NPCs rather than embryonic NPCs, ensuring a standardized, reproducible model system. Since EpoR expressions tended to be reduced in adult NPCs, the Epo-effect in embryonic NPCs may show stronger mitogenic responses to Epo due to higher EpoR expression levels. Confirmatory experiments with fetal rat NPCs are likely to support our data.

## Conclusion

We demonstrated, for the first time, an upregulation of EpoR expression in neuroplacodes of OSD, and the neuroprotective effects of Epo when added to aAF-treated NPC cultures. These results suggest that Epo is a promising mediator and its receptor is a suitable target for surgery accompanying neuroprotective therapies in OSD.

## Supplementary Information

Below is the link to the electronic supplementary material.

Supplementary file1 (PDF 650 KB)

Supplementary file2 (PNG 1464 KB)

## Data Availability

No datasets were generated or analysed during the current study.

## Abbreviations

aAF: Artificial amnionic fluid
Epo: Erythropoietin
EpoR: Erythropoietin receptor
Hif: Hypoxia inducible factor
IF: Immunofluorescence
IR: Immunoreactivity
OSD: Open spinal dysraphism (summarizes myelomeningocele and myeloshisis)
MMC: Myelomeningocele
NPC: Neural progenitor cells
SC: Spinal cord
RA: Retinoic acid
RA-OSD model: Retinoic acid-induced OSD model
qRT-PCR: Quantitative reverse transcription-polymerase chain reaction
