# Supplementary figures and images for: Neuroprotective properties of erythropoietin (Epo) and its receptor (EpoR) in open spinal dysraphism (OSD): an investigation of EpoR expression in a rat OSD model, along with in vitro studies on the neuroprotective effects of Epo on rat spinal cord-derived neural progenitor cells
Source: Childs Nerv Syst. 2025 Nov 17;41(1):358. doi: 10.1007/s00381-025-07032-8 (PMC12620314; doi:10.1007/s00381-025-07032-8)

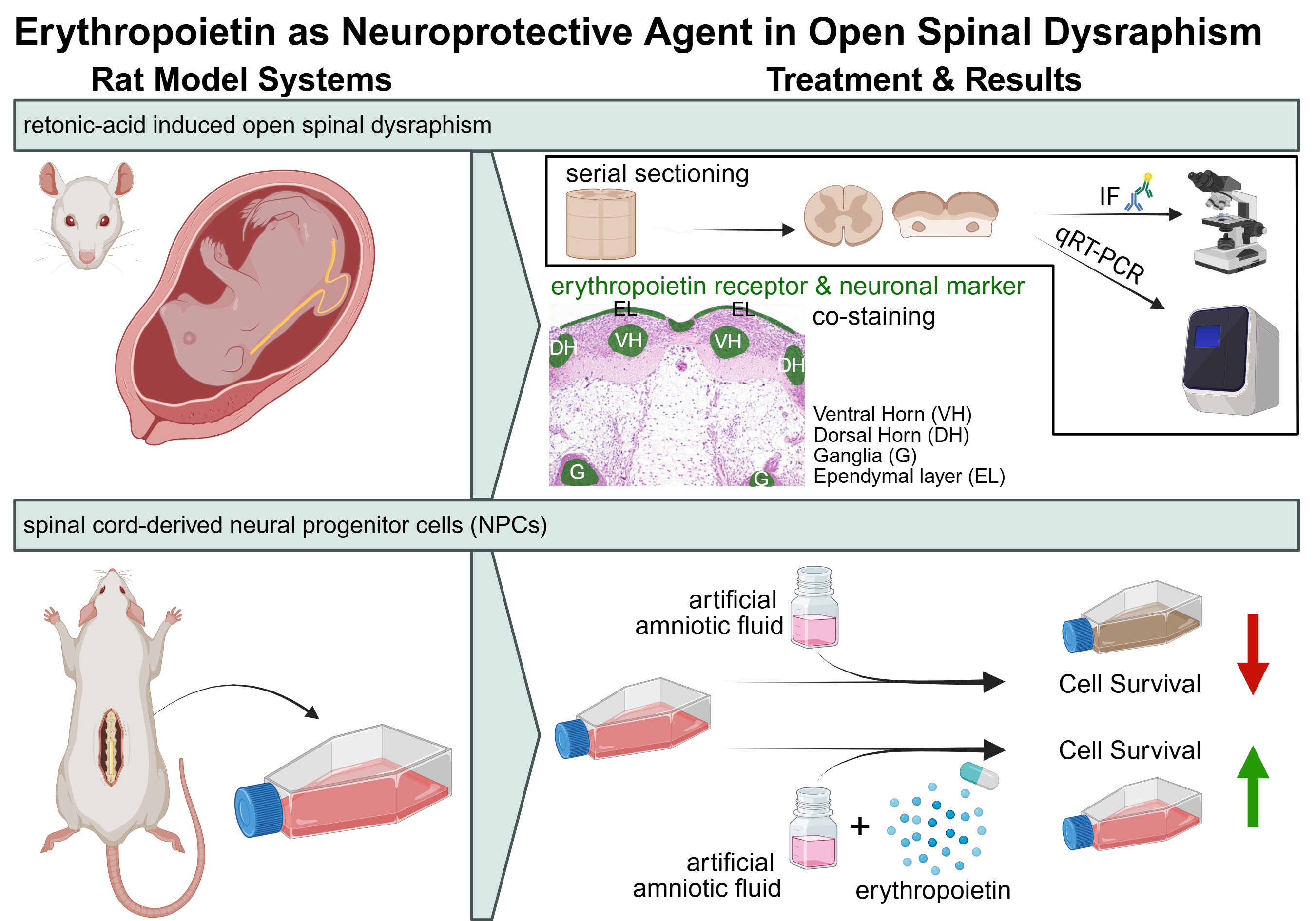

Supplement: Supplementary file 2 — Supplementary file2 (PNG 1464 KB) [file 381_2025_7032_MOESM2_ESM.png]
